# E242-E261 region of MYC regulates liquid-liquid phase separation and tumor growth *by* providing negative charges

**DOI:** 10.1016/j.jbc.2024.107836

**Published:** 2024-09-28

**Authors:** Xiaoying Pei, Yatao Chen, Linjing Liu, Li Meng, Jun Zhang, Yan Liu, Liming Chen

**Affiliations:** 1 Department of Biochemistry, School of Life Sciences, Nanjing Normal University, Nanjing, China; 2 Department of Orthodontics, Affiliated Hospital of Stomatology, Nanjing Medical University, Nanjing, China; 3 Jiangsu Institute of Cancer Research, Jiangsu Cancer Hospital, The Affiliated Cancer Hospital of Nanjing Medical University, Nanjing, China

**Keywords:** MYC, liquid-liquid phase separation, multivalent electrostatic interactions

## Abstract

MYC is one of the most extensively studied oncogenic proteins and is closely associated with the occurrence and progression of many tumors. Previous studies have shown that MYC regulates cell fate through its liquid-liquid phase separation mechanism, which is dependent on two disordered domains within its N-terminal transcriptional activation regions. In this study, we revealed that the negatively charged conserved region (E242-E261) of the MYC protein controls its condensation formation and irreversible aggregation through multivalent electrostatic interactions. Furthermore, deletion or mutation of the E242-E261 amino acids in the MYC protein enhances the transcriptional function of MYC by altering its aggregation capacity and subsequently promoting cancer cell proliferation. The discovery of the negatively charged region and its regulatory action on the phase separation of MYC provides a new understanding of the aggregation and function of MYC.

LLPS is an intrinsic physiological phenomenon characterized by the emergence of a concentrated phase, typically manifested as liquid droplets, that coexists with a more dilute phase (1, 2). This process is fundamental to the assembly of a variety of biomolecules, including proteins and nucleic acids, into functional condensates that facilitate the efficient execution of biochemical reactions (3). Proteins involved in LLPS are often characterized by a sequence of low-complexity and high charge polarity, conferring distinct hydrophobic and electrostatic interactions that are essential for driving LLPS((4)). LLPS has been implicated in the initiation of protein aggregation, which in turn has the potential to precipitate pathological aggregates in a spectrum of diseases, including cancer (5, 6, 7). Several oncogenic proteins, including transcription factors, have been observed to undergo LLPS both in cellular environments and under *in vitro* conditions (8, 9).

The *MYC* oncogene plays a central role in tumorigenesis by encoding an important transcription factor that regulates a large number of genes (10, 11, 12). Overexpression of MYC is a common feature across various cancers, particularly in aggressive subtypes such as triple-negative breast cancer (13). However, the intrinsically disordered nature of MYC poses a significant challenge for therapeutic targeting (14, 15, 16). Recent studies have indicated that MYC can multimerize *in vivo* and thereby promote the repair of transcription-associated DNA damage, a process that is modulated by ubiquitination (17). Despite these advances, the specific role of electrostatic interactions in MYC condensation and the key domain responsible for driving its aggregation have yet to be elucidated. In this study, we reveal that a specific segment of the MYC protein, E242-E261, is crucial for its condensation and aggregation, thereby enhancing its transcriptional activity. Our findings shed light on the underlying mechanisms of MYC aggregation and may have significant implications for the development of novel therapeutic interventions targeting MYC.

## Results

### E242-E261 regulates the condensation and aggregation of the MYC protein

**Figure 1:**
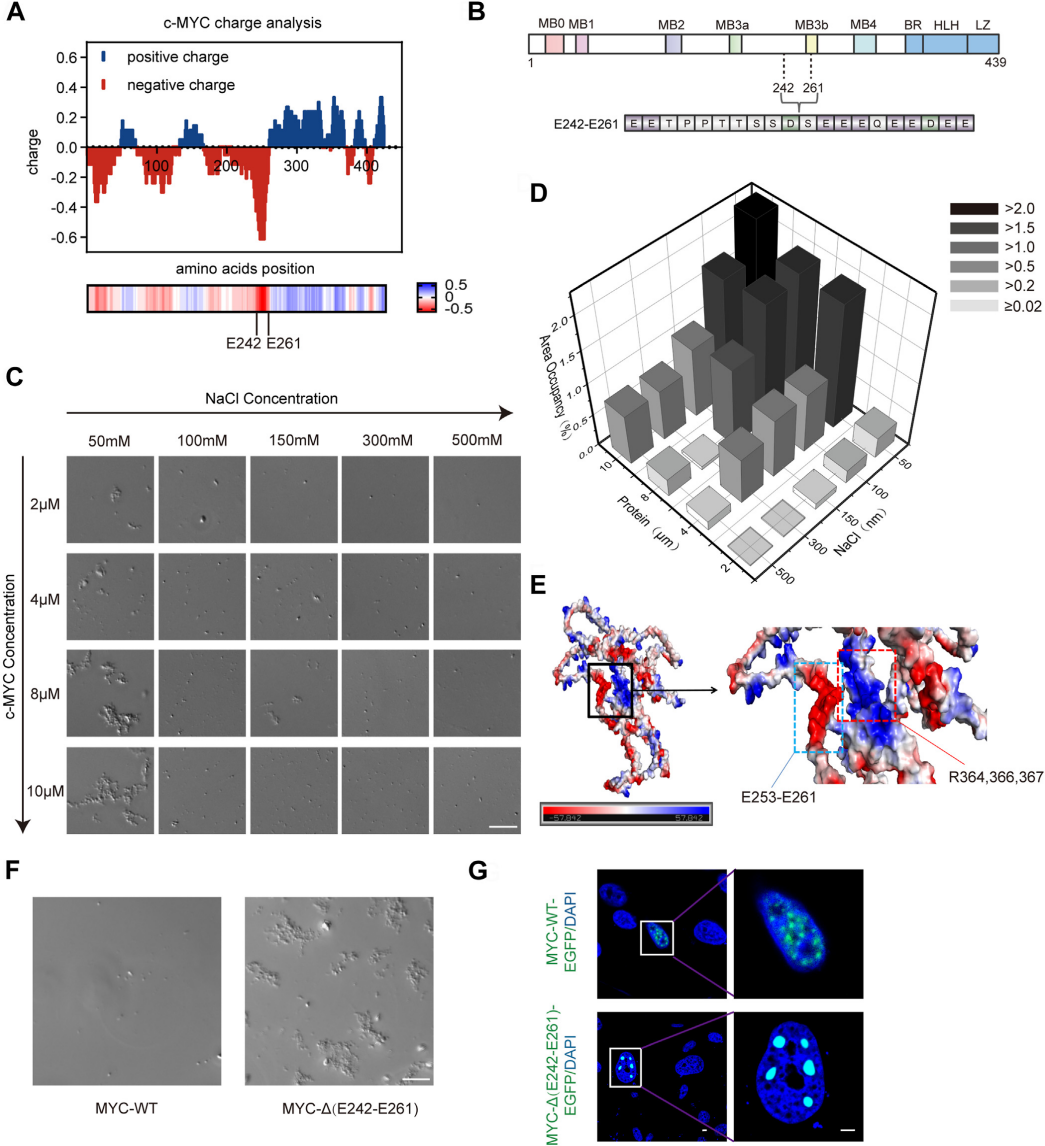
**E242-E261 regulates LLPS of MYC protein through MEIs.***A*, average charge analysis of the MYC protein amino acid sequence. *Red*, negatively charged; *blue*, positively charged. *B*, amino acid composition of E242-E261 domain. MB: MYC boxes. BR: the basic region. HLH: helix-loop-helix. LZ: leucine zipper. *C*, MYC condensation *in vitro* dependent NaCl and protein concentration. Scale bar, 10 μm. *D*, quantitative comparison of phase condensation of MYC with different NaCl and protein concentrations. *E*, predicted 3D structure of MYC protein by Alphafold2. *Red*, negatively charged; *blue*, positively charged. *F*, droplet formation assay *in vitro* for 10 μM MYC-WT and its truncation in 150 mM NaCl. Scale bar, 10 μm. *G*, representative images of MDA-MB-468 cells with overexpression of indicated EGFP-MYC-WT and EGFP-MYC-Δ(E242-E261). Scale bar, 10 μm.

To investigate the mechanism of MYC granule formation, we performed a structural analysis of the MYC protein. The protein is composed of a disordered N-terminal segment and a C-terminal basic region helix-loop-helix leucine zipper (bHLHLZ) domain, which is predominantly responsible for its transcriptional activity. Our investigation revealed that the C-terminal domain (CTD) of MYC is positively charged, in contrast to the segment spanning amino acids 242 to 261, which is predominantly negatively charged (Fig. 1*A*). The proteins that rely on multivalent electrostatic interactions (MEIs) to support condensation are known to be sensitive to changes in salt concentration, for that high concentration of salt ions can shield the electrostatic interactions and dissolve the particles (18). We hypothesized that the condensation of MYC is governed by MEIs and that the E242-E261 segment of MYC is critical for its LLPS (Fig. 1*B*).

To test the above mentioned hypothesis, we purified the full-length MYC protein (FL-MYC) and evaluated its condensation capacity under a range of salt conditions (500, 300, 150, 100, 50 mM NaCl). Microscopic examination revealed that the propensity of FL-MYC for condensation decreased with increasing salt concentration, underscoring the critical role of MEIs in MYC protein condensation (Fig. 1, *C* and *D*). To ascertain the importance of the E242-E261 segment for MYC condensation, we utilized the Alphafold2 platform to predict the three-dimensional structure of MYC and identified that the E242-E261 segment is exposed and capable of engaging in interactions with other MYC molecules under certain conditions (Fig. 1*E*). We then constructed a deletion mutant of MYC lacking the E242-E261 segment (MYC-ΔE242-E261), expressed it in *Escherichia coli*, and assessed its phase separation capabilities. Remarkably, the granule-forming capacity of MYC was significantly augmented in the absence of the E242-E261 segment, even under identical salt and protein concentrations (Fig. 1*F*). Consistent with the enhanced particle clustering observed *in vitro* upon E242-E261 deletion, we also observed a substantial increase in the size of MYC particles *in vivo* within MDA-MB-468 cells transfected with an EGFP-tagged E242-E261 deletion mutant (EGFP-MYC-ΔE242-E261) compared to EGFP-MYC-WT (Fig. 1*G*). Collectively, our data indicate that the deletion of E242-E261 promotes MYC granule clustering tendency *via* MEIs.

To explore the differences in the characteristics of the particles formed by the MYC-ΔE242-E261 mutant compared to wild-type MYC, we subjected them to temperature cycling and monitored the formation and dissolution of droplets *in vitro* (Fig. S1). The purified wild-type MYC displayed a scarcity of droplets at low temperatures (4 °C), but as the temperature increased to 37°C, its propensity for LLPS markedly enhanced, resulting in the formation of a modest number of condensates. Importantly, this condensation was reversible. Upon cooling back to 4°C, the condensates spontaneously disintegrated, and the capacity for LLPS was restored to a reduced level. In contrast, the MYC-ΔE242-E261 mutant exhibited irreversible aggregation. Although the aggregation intensified with temperature elevation, these aggregates persisted even when the temperature was lowered. These findings collectively demonstrate that the removal of the strong negative charge from this domain, which modulates inter- and intramolecular MEIs, endows the MYC-ΔE242-E261 protein with the ability to form aggregates that undergo irreversible changes with temperature, thus exhibiting a thermal stability distinct from the liquid-like droplets of wild-type MYC. Moreover, it is intriguing to note that conditions that either favor or hinder LLPS also influence protein aggregation, suggesting the potential for an LLPS-mediated aggregation pathway for MYC.

### E242-E261-mutation shows enhanced oncogenicity

**Figure 2:**
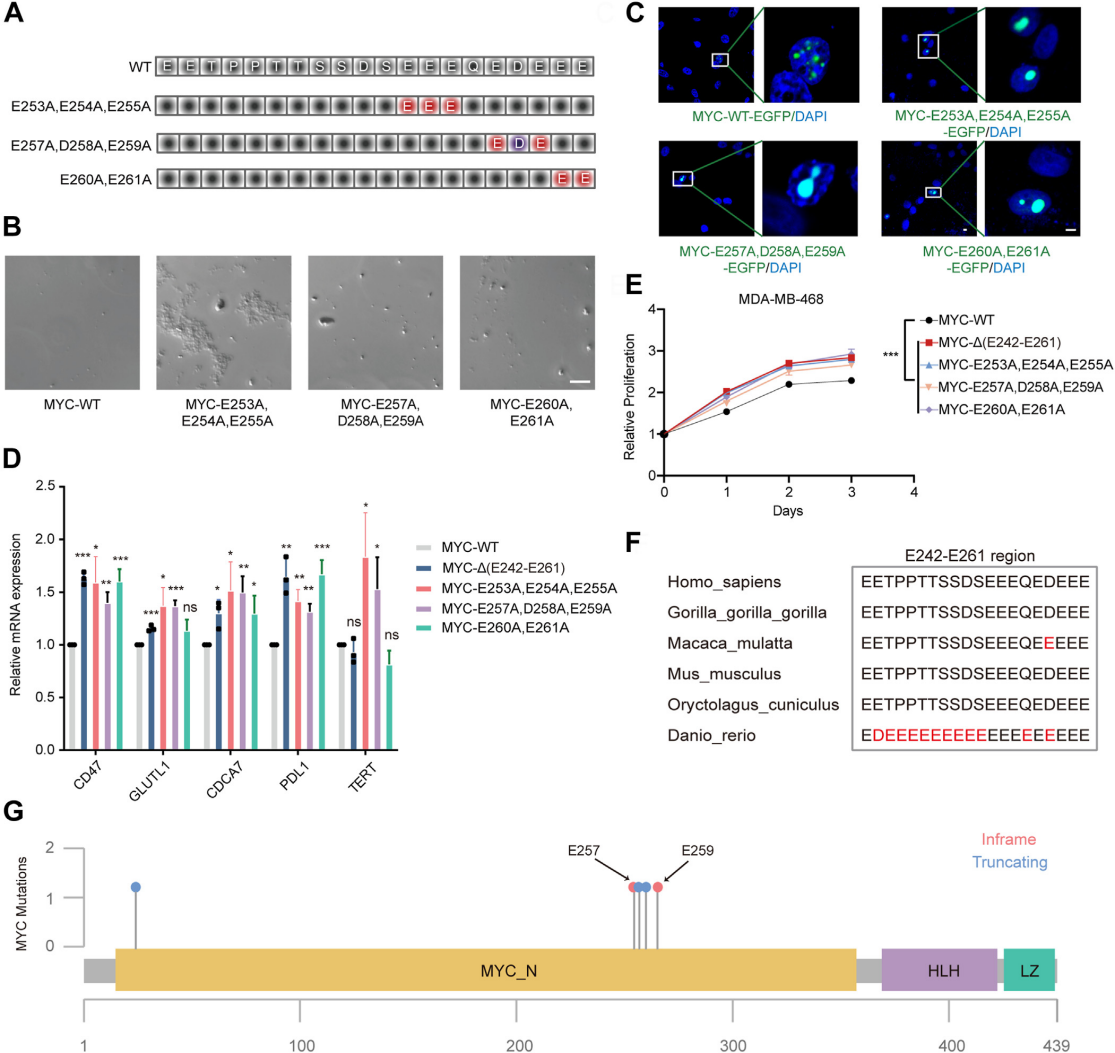
**E242-E261-mutation shows enhanced oncogenicity**. *A*, schematic diagram of the MYC mutants. *B*, droplet formation assay *in vitro* for MYC mutants. Scale bar, 10 μm. *C*, representative images of MDA-MB-468 cells with overexpression of indicated MYC mutants. Scale bar, 10 μm. *D*, qRT-PCR analysis of transcriptional levels of MYC target genes in MDA-MB-468 cells (n = 3; mean ± SEM; two-tailed *t* test). *E*, growth potential of MDA-MB-468 cells with overexpression expression of indicated MYC wild type or mutated genes *in vitro* by Cell Counting Kit-8 proliferation assay. Data are mean ± SD. ∗∗∗, *p* < 0.001 (*t* test). *F*, protein sequence alignment of multi-species MYC using ClustalW. Inside the *black frame*, E242-E261 domain. *G*, a diagram summarizing the MYC mutations identified in clinical breast cancer samples. *Blue dots*, MYC truncating mutations. *Pink dots*, MYC inframe mutations.

Building on our previous discoveries, we delved deeper to identify the specific amino acids within the E242-E261 segment of MYC that might be responsible for its aggregation properties, as the strong negative charge in this region could be attributed to a series of consecutive glutamic and aspartic acids. To this end, we generated three different mutants, each designed to neutralize the charge polarity through targeted mutations: the MYC-E253A, E254A, E255A mutant, the MYC-E257A, D258A, E259A mutant, and the MYC-E260A, E261A mutant (Fig. 2*A*). In line with the findings for MYC-ΔE242-E261, we observed the formation of large, irregular aggregation entities in the MYC-E253A, E254A, E255A mutant, whereas an increased propensity for LLPS *in vitro* was observed in the MYC-E257A, D258A, E259A mutant and the MYC-E260A, E261A mutant (Fig. 2*B*). As expected, the size of MYC puncta was found to be enlarged in these three mutants when expressed in MDA-MB-468 cells (Fig. 2*C*). In addition, we extended our investigation to evaluate the particle morphology for each mutant upon knockdown of endogenous MYC expression (Fig. S2, *A*–*C*).

Recognizing the role of MYC as a transcription factor that modulates a number of biological functions, we proceeded to validate the expression of its target genes using RT-qPCR in MDA-MB-468 cells transfected with the various MYC mutants. Genes known to be regulated by MYC, such as CD47, GLUT1, CDCA7, PDL1, and TERT, were confirmed to be upregulated upon transfection with the MYC mutants (Fig. 2*D*). Similar results were obtained after the knockdown of endogenous MYC and subsequent transfection with MYC-ΔE242-E261 (Fig. S2*D*). In MDA-MB-468 cells, all MYC mutants were found to enhance cell proliferation compared to wild-type MYC (Fig. 2*E*), suggesting that alterations at the E242-E261 site may augment the oncogenic potential of MYC. Moreover, we discovered that the E242-E261 segment of MYC is evolutionarily conserved among chordates, as evidenced by multiple sequence alignment analysis, further supporting the importance of this region for MYC functions related to aggregation and LLPS (Fig. 2*F*).

## Discussion

Liquid-liquid phase separation (LLPS) of biomolecules, such as proteins and nucleic acids, is now recognized as a ubiquitous mechanism underlying the formation of membraneless organelles. These concentrated compartments, segregated from the cytoplasm, are essential for a variety of cellular processes. They not only facilitate essential cellular functions but also serve as sites for macromolecular isolation and storage, as well as for cell signaling and communication (19). The driving forces behind LLPS are predominantly multivalent interactions, including both electrostatic and hydrophobic interactions (20).

Several proteins, including FUS (21), TDP433 (22), BRD4 (23), SHP2 (24), LAF-1 (25), Ddx4 (18), hnRNPA1 (26), and SPOP (27), have been observed to exhibit significant variations in their condensation abilities in response to different salt ion concentrations. This underscores the predominant role of electrostatic interactions in protein condensation, especially under high salt conditions. Interestingly, much of the current research has focused on the amino acid sequences within proteins themselves. For instance, the removal or alteration of the arginine-rich regions in FUS or the positively charged PTP domain in SHP2 has been shown to diminish the LLPS capacity of these proteins. Conversely, phosphorylation of serine or tyrosine residues in Tau has been shown to enhance protein aggregation (28). These findings suggest that different charged amino acids may have different or even opposing effects on protein aggregation. In our study, we focused on the MYC protein and discovered that the E242-E261 region plays an inhibitory role in the formation of MYC LLPS formation and aggregation. Deletion or mutation of this region results in an enhanced aggregation behavior of MYC. While the importance of the intrinsically disordered region (IDR) of MYC for LLPS has been previously reported, few studies have highlighted the importance of regions with accumulated negative charge for LLPS. Notably, despite a low mutation frequency, several spontaneous mutation sites within the E242-E261 region have been identified in clinical breast cancer samples. Future research is crucial to further clarify the interplay between the physicochemical properties of protein amino acid sequences and LLPS.

As a transcription factor, MYC relies on LLPS to form nuclear condensed particles that modulate transcriptional output (23). Our findings indicate that the increased cohesion due to the deletion or mutation of the E242-E261 region in MYC correlates with increased expression of its target genes and enhanced tumor cell proliferation. This suggests a direct link between phase separation and the functional activity of MYC. In addition, some studies have reported a protective role of MYC nuclear condensation in the genome (17). The pronounced increase in nuclear puncta observed in our MYC E242-E261 mutant data may also contribute to genomic protection, potentially influencing the favorable selection processes in tumor growth.

## Conclusion

In summary, there is compelling evidence indicating that the neutralization of the charge polarity within the E242-E261 of the MYC segment facilitates the formation of pathological MYC aggregates, thereby accelerating tumor growth. Deletion or mutation of the charged amino acids at this locus intensifies the aggregation process of MYC. The E242-E261 region, rich in consecutive glutamic and aspartic acids, relies on the charge polarity of these residues, which are likely crucial for maintaining MYC's intermolecular and intramolecular interactions.

## Experimental procedures

### Cell culture

The human breast cancer cell line MDA-MB-468 was cultivated in Dulbecco's Modified Eagle's Medium (DMEM), enriched with 10% Fetal Bovine Serum (FBS) and 1% Penicillin/Streptomycin. The culture was maintained at a temperature of 37 °C within an incubator calibrated to a 5% CO2 atmosphere.

### Cell viability assay

MDA-MB-468 cells were initially seeded into a 6-well culture plate and subsequently transfected with the MYC variants. Following a 2-day transfection period, the cells were enumerated and aliquoted into a 96-well plate, with each condition being replicated thrice. To assess cell viability, the CCK-8 assay kit from Dojindo Laboratories, was employed, with the procedure strictly adhering to the manufacturer's guidelines.

### Plasmids construction and transfection

MYC-Δ(E242-E261)-mEGFP, MYC-E253A,E254A,E255A-mEGFP, MYC-E257A,D258A,E259A-mEGFP, MYC-E260A, E261A-mEGFP variant mutants and MYC-mEGFP were transfected into MDA-MB-468 cells using Lipofectamine 3000 (Invitrogen) according to the product brochures. The transfected cells were harvest for protein lysis after 48 h. The MYC-mEGFP constructs were generated by PCR and subcloned into the pmEGFP-N2 mutated by pEGFP-N2 (Clontech). MBP-MYC constructs were generated by PCR and subcloned into the pET-MBP-3C vector. The MYC-Δ(E242-E261)-mEGFP constructs were generated by gene splicing by overlap extension PCR (SOEPCR) and subcloned into the pmEGFP-N2. The MYC-E253A,E254A, E255A-mEGFP, MYC-E257A,D258A, E259A-mEGFP, MYC-E260A, E261A-mEGFP constructs were generated by site-directed mutagenesis and subcloned into the pmEGFP-N2. In the siRNA-mediated knockdown experiment targeting endogenous MYC, followed by the transfection of MYC mutants, all mutant plasmids were generated by site-directed mutagenesis and subcloned into the pmEGFP-N2. Primer sequences are listed in Table S1.

### RNAi

The siRNA transfections were carried out with Lipofectamine RNAi-MAX (Thermo Fisher, catalog no. 13778100) in the antibiotic-free medium according to the manufacturer’s instructions. The sequences for control siRNA were the sense(5-UUC UCC GAA CGU GUC ACG UTT-3) and antisense(5-ACG UGA CAC GUU CGG AGA ATT-3). The sequences for MYC siRNA were: 1) sense (5-CGA UGU UGU UUC UGU GGA A-3) and antisense (5-UUC CAC AGA AAC AAC AUC G-3); and 2) sense (5-GGA ACU AUG ACC UCG ACU A-3) and antisense (5-UAG UCG AGG UCA UAG UUC C-3); and 3) sense (5-CCA AGG UAG UUA UCC UUA A-3) and antisense (5-UUA AGG AUA ACU ACC UUG G-3).

### Protein expression and purification

Plasmids containing genes tagged with His and MBP were transformed into *E. coli* (*E. coli*) BL21 cells. All proteins were expressed in *E. coli* BL21 (DE3) Rosetta cells. The cells were cultured at 37 °C in LB media with 100 mg/ml Ampicillin until they reached OD 0.6 to 0.8. After that, *E. coli* cells expressing protein were induced with 0.1-0.3 mM IPTG at 16 °C for 12 h before being sonicated in a lysis buffer consisting of 50 mM Tris-HCl pH 7.5, 500 mM NaCl, and 1 mM PMSF. The proteins underwent initial purification *via* Ni affinity chromatography, followed by further purification using Superdex 200 Increase 10/300 Gl. They were then stored in a solution of 50 mM Tris-HCl pH 7.5, 500 mM NaCl, 1 mM DTT, 10% glycerol at a temperature of −80 °C. The purified proteins were analyzed utilizing SDS-PAGE, followed by Coomassie blue staining. Protein concentration quantification was accomplished using Nanodrop measurement for OD562 and validated by comparing against Coomassie blue staining of BSA with known concentrations.

### High content image

MDA-MB-468 cells expressing MYC-mEGFP and its variants were seeded onto a 24-well glass bottom plate (Sofa P24–1.5H-N) and imaged using the NIS-element cell imaging analysis system (Leica 710) with a 40× objective lens applied in each condition. Data were analyzed using images collected from 10 representative fields in each group. Single cells were identified using DAPI as a reference, while spot quantification was conducted based on the area and intensity of the spots detected *via* the mEGFP channel.

### *In vitro* droplets formation and turbidity assays

For the droplet formation assay, the MBP-MYC and its variant proteins, which had been purified, were combined with a buffer comprising 50 mM Tris pH 7.4. Following the addition of proteinase 3C, the mixture was incubated for 2 h at room temperature. Proteinase 3C cleaves the MBP-tag present in the N terminal. Finally, 10 μl of each sample was pipetted onto a glass dish and subsequently imaged using a Nico TIAR microscope.

### RT-qPCR

Total RNA was extracted from the cells using the Total RNA Isolation Kit (Beibei biotechnology, 082,001). After quantification using a Nanodrop spectrophotometer (Thermo Scientific), 1000 ng of total RNA was reverse-transcribed into cDNA with the cDNA Reverse Transcription Kit (Thermo Scientific, 4,374,966). Following this, quantitative PCR was conducted using SYBR Green Realtime PCR Master Mix (Vazyme) and quantified *via* the StepOne Real-Time PCR System (Bio-Rad). The amplification parameters were set at 95 °C for 3 min followed by 10 s at 95 °C and 30 s at 60 °C for a total of 40 cycles. β-actin was utilized as an internal control and primer sequences are listed in Table S1.

### Quantification and statistical analysis

All the experiments were repeated at least three times, and the statistical details of experiments can be found in the figures and legends. All comparisons were performed in GraphPad Prism9.0. (ns, not statistically significant, ∗*p* < 0.05, ∗∗*p* < 0.01, ∗∗∗*p* < 0.001).

## Data availability

All data used in this study have been presented in the manuscript and supporting information.

## Supporting information

This article contains supporting information.

## Conflict of interest

The authors declare that they have no conflicts of interest with the contents of this article.
