# Supplementary material for: E242-E261 region of MYC regulates liquid-liquid phase separation and tumor growth by providing negative charges
Source: J Biol Chem. 2024 Sep 28;300(11):107836. doi: 10.1016/j.jbc.2024.107836 (PMC11530832; doi:10.1016/j.jbc.2024.107836)
Supplement: Supporting Information [file mmc1.docx]

**Supporting Material**

**E242-E261 region of MYC regulates liquid-liquid phase separation and tumor growth via providing negative charges**

Xiaoying Pei^1^, Yatao Chen^1^, Linjing Liu^1^, Li Meng^2^, Jun Zhang^1,*^, Yan Liu^1,*^, Liming Chen ^1,3,*^

^1^ Department of Biochemistry, School of Life Sciences, Nanjing Normal University, Nanjing, China

^2^ Department of Orthodontics, Affiliated Hospital of Stomatology, Nanjing Medical University, Nanjing 210029, China.

^3^Jiangsu Institute of Cancer Research, Jiangsu Cancer Hospital, the Affiliated Cancer Hospital of Nanjing Medical University, Nanjing, China

*Correspondence: Jun Zhang: 171201024@njnu.edu.cn; Yan Liu: [llliuyan@sina.com;](mailto:llliuyan@sina.com;) Liming Chen: chenliming1981@njnu.edu.cn;

**Supporting Information Figures**

**
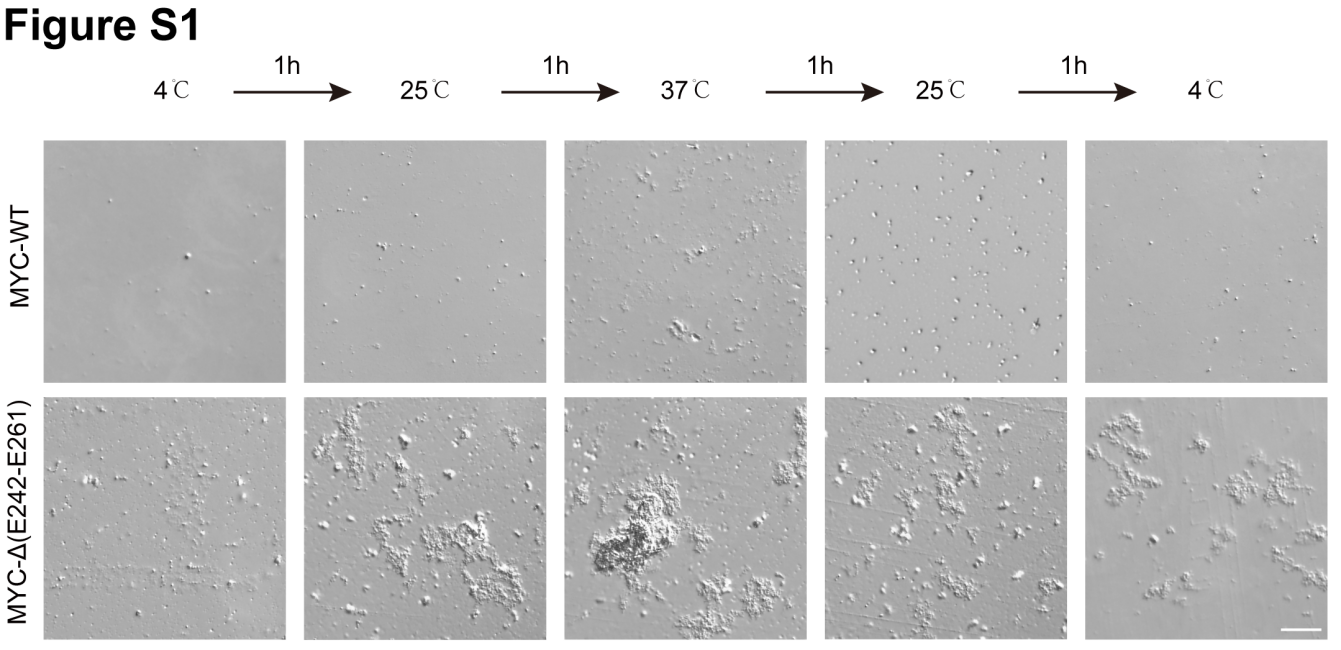
**

**
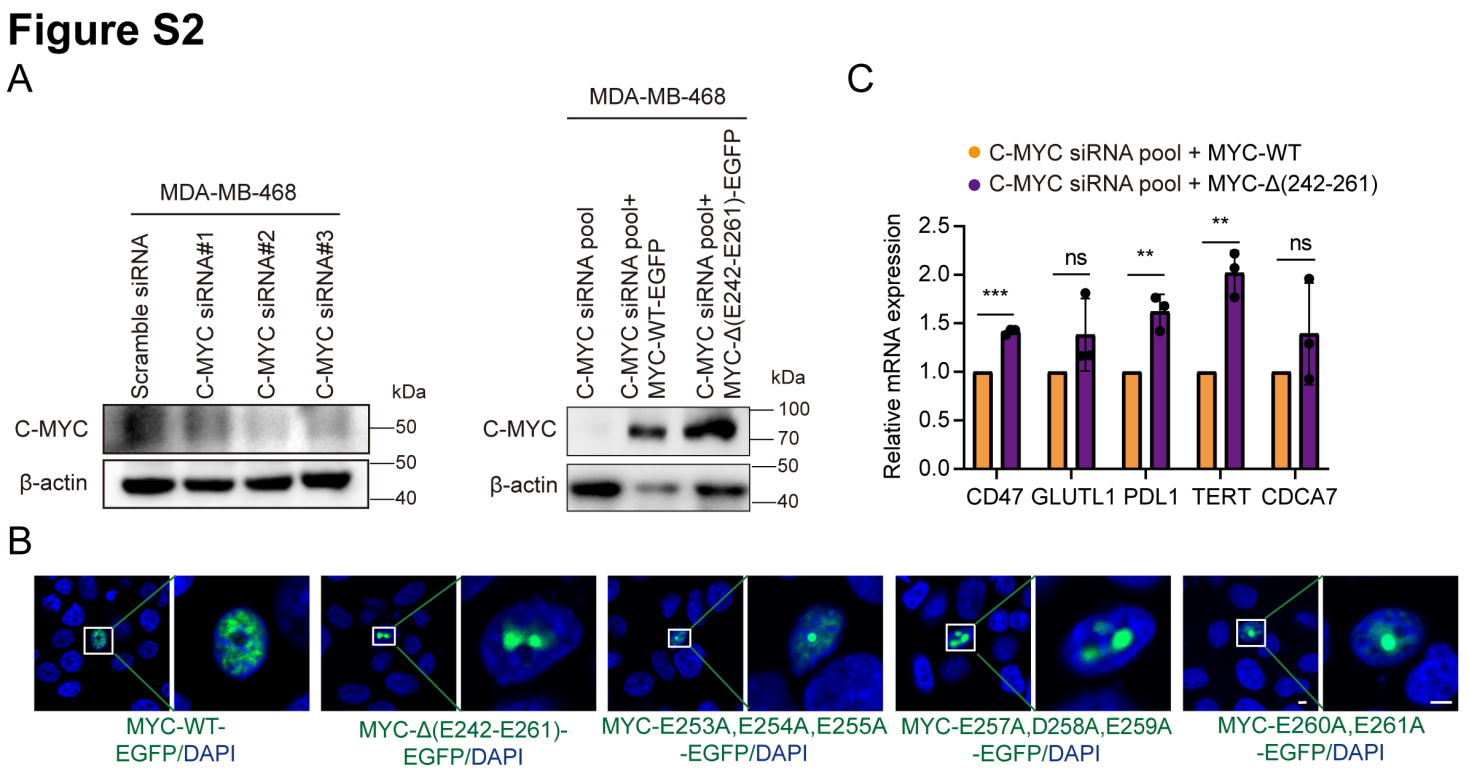
**

**Supporting Information Figure legends**

**Figure S1. E242-E261 regulates the condensation and aggregation of the MYC protein. (A)** Quantitative comparison of droplet formation assays conducted *in vitro* for 1μM MYC-WT and its truncation in 150mM NaCl at varying temperatures. Scale bar, 10μm.

**Figure S2. E242-E261-mutation shows enhanced oncogenicity. (A-B)** Western blotting analysis of MYC diminished in MDA-MB-468 cells and MDA-MB-468 cells transfected siRNA pool (simultaneous transfection with siRNA#1,2,3) followed by transfection with exogenous MYC. To prevent the exogenous MYC we subsequent transfected from also being knocked down by the siRNAs, three amino acids in the siRNA targeting sequence were subjected to synonymous mutation for the plasmids. **(C)** Representative images of MDA-MB-468 cells with overexpression of the indicated mutants after knockdown of endogenous MYC. Scale bar, 10μm. **(D)** qRT-PCR analysis of the transcriptional levels of MYC target genes in MYC-depleted MDA-MB-468 cells with exogenous MYC mutants (n = 3; mean±SD; two-tailed *t* test). ***, *p* < 0.001. **, *p* < 0.01.

**Supporting Information Tables**

**Table S1. primers and siRNAs used in this study**

| primers | sequence |
| --- | --- |
| β-actin-F | 5' -AGAGCTACGAGCTGCCTGAC-3' |
| β-actin-R | 5' -AGCACTGTGTTGGCGTACAG-3' |
| CD47-F | 5' -TGAGTCTCTGTATTGCGGCG-3' |
| CD47-R | 5' -AGTCTTCTGATTGGAAGCCACA-3' |
| GLUT1-F | 5' -GAACTCTTCAGCCAGGGTCC-3' |
| GLUT1-R | 5' -ACCACACAGTTGCTCCACAT-3' |
| PDL1-F | 5' -TGGCATTTGCTGAACGCATTT-3' |
| PDL1-R | 5' -AGTGCAGCCAGGTCTAATTGT-3' |
| TERT-F | 5' -CGAAAACCTTCCTCAGGACCC-3' |
| TERT-R | 5' -GGCCGGCATCTGAACAAAAG-3' |
| CDCA7-F | 5' -GACCATTGTGGATTTTTACAGAAAC-3' |
| CDCA7-R | 5' -TGACTGCAGCCTCATTCCAT-3' |
| siRNA-1-mut-F | 5' -ATGAGGAAGAACGATGTGGTGTCCGTGGAAAAGAGGCAG-3' |
| siRNA-1-mut-R | 5' -CTGCCTCTTTTCCACGGACACCACATCGTTCTTCCTCAT-3' |
| siRNA-2-mut-F | 5' -TTCACCAACAGGAACTACGATCTGGACTACGACTCGG-3' |
| siRNA-2-mut-R | 5' -CCGAGTCGTAGTCCAGATCGTAGTTCCTGTTGGTGAA-3' |
| siRNA-3-mut-F | 5' -GAAAAGGCCCCCAAGGTTGTAATACTTAAAAAAGCCACA-3' |
| siRNA-3-mut-R | 5' -TGTGGCTTTTTTAAGGATAACTACCTTGGGGGCCTTTTC-3' |
